# Shikonin induces apoptosis and inhibits migration of ovarian carcinoma cells by inhibiting the phosphorylation of Src and FAK

**DOI:** 10.3892/ol.2014.2771

**Published:** 2014-12-05

**Authors:** ZHENFENG HAO, JING QIAN, JISHI YANG

**Affiliations:** 1 Laboratory of Traditional Chinese Medicines, Medical College of Yangzhou University, Yangzhou, Jiangsu 225001, P.R. China; 2 Department of Gynecology and Obstetrics, The Affiliated Taixing Hospital, Yangzhou University, Yangzhou, Jiangsu 225004, P.R. China

**Keywords:** shikonin, ovarian cancer, apoptosis, Src, focal adhesion kinase, phosphorylation

## Abstract

The present study identified that shikonin, a naphthoquinone extracted from the roots of *Lithospermum erythrorhizon*, inhibits the migration of ovarian cancer cells and induces their apoptosis by impairing the phosphorylation of two kinases, proto-oncogene tyrosine protein kinase Src (Src) and focal adhesion kinase (FAK). Ovarian carcinoma SKOV-3 cells were treated with various concentrations of shikonin and analyzed for the effects on cell migration, invasion and apoptosis via Transwell assays and flow cytometry. In addition, the effects of shikonin administration on the expression and phosphorylation of Src and FAK in the SKOV-3 cells were analyzed by western blotting. Shikonin appeared to induce apoptosis and decrease cell migration in the SKOV-3 ovarian cells. Furthermore, the present study provides evidence that shikonin may exert these effects on human ovarian carcinoma cells via the inhibition of the protein tyrosine kinases, Src and FAK. Thus, shikonin should be considered for additional investigation as a candidate agent for the prevention and treatment of human ovarian cancer.

## Introduction

Shikonin, a naphthoquinone extracted from the roots of *Lithospermum erythrorhizon*, is a potent antioxidant with anti-inflammatory, antiviral and cancer-preventing properties (1). Shikonin exhibits significant cytotoxic activity against multiple cancer cell types *in vitro* and *in vivo* (2,3), and a number of studies have previously established a potential role for shikonin as a candidate therapeutic agent in the treatment of cancer (4,5). However, the mechanism by which shikonin achieves this effect has yet to be fully elucidated (6).

Ovarian carcinoma is the most lethal type of gynecological malignancy. The response to traditional platinum-based chemotherapy is poor in numerous patients, therefore, current research is focused on the development of novel therapeutic strategies. Protein tyrosine kinases (PTKs) are important in cellular signal transduction pathways and regulate numerous cellular activities, including cell growth, migration, differentiation and apoptosis (7,8). Furthermore, the abnormal activation of PTKs is closely associated with ovarian carcinoma (8), therefore, PTKs are attractive targets for anticancer agents.

The expression and activity of the proto-oncogene tyrosine kinase Src (Src) is associated with a poor prognosis and advanced malignancy in a range of types of human cancer, including ovarian carcinoma (9,10). Focal adhesion kinase (FAK), an intracellular PTK recruited to focal adhesion sites, acts via cell surface receptors as a major mediator of signal transduction (11). FAK has been demonstrated to be key factor in the regulation of cell survival (12), proliferation, differentiation, migration, invasion (13) and angiogenesis (14), all of which are vital processes in the development of cancer. Furthermore, FAK is overexpressed in ovarian cancer (15). Therefore, FAK may be involved in promoting tumorigenesis and metastasis in cancer.

In the present study, it was hypothesized that shikonin may have a role as an inhibitor of ovarian cancer cells growth and migration, and therefore, could potentially serve as a therapeutic agent for the management of human ovarian cancers.

## Materials and methods

### Preparation of shikonin

Shikonin was purchased from ChromaDex, Inc., (cat. no., ASB-00019210-005; Irvine, CA, USA), dissolved in dimethyl sulfoxide (DMSO; Sigma-Aldrich, St. Louis, MO, USA) and stored at −20°C. For all experiments in the present study, the final concentrations of the compounds analyzed were prepared by diluting the stock solution with culture medium, while the control cultures were diluted with the carrier solvent (0.1% DMSO).

### Cell culture

SKOV-3 cells were purchased from the American Type Culture Collection (Manassas, VA, USA) and maintained in a monolayer culture at 37°C and 5% CO_2_ in McCoy’s 5A medium (Gibco Life Technologies, Carlsbad, CA, USA) with 10% fetal bovine serum (Gibco Life Technologies).

### Cytotoxicity assay

The cytotoxic effect of shikonin on the SKOV-3 cells was measured by performing a Cell Counting kit (CCK)-8 assay (Dojindo Laboratories, Kumamoto, Japan). Briefly, the cells were dispensed into a 96-well flat-bottomed microtiter plate (Thermo Scientific Nunc, Roskilde, Denmark) at a density of 1×10^4^ cells/well, followed by treatment with various concentrations of shikonin (1, 2, 4, 8, 16, 32, 64, 128 or 256 μM) for 48 h. Cell growth was measured using an enzyme-linked immunosorbent assay reader (Tecan Spectra, Wetzlar, Germany) to analyze the CCK-8 assay.

### Flow cytometric analysis

The rate of apoptosis was measured using an Annexin V-fluorescein isothiocyanate/propidium iodide (FITC/PI) apoptosis detection kit (Invitrogen Life Technologies, Carlsbad, CA, USA), according to the manufacturer’s instructions. The cells were exposed to various concentrations of shikonin (0, 4, 8 and 16 mmol/l), incubated for 48 h, collected and washed twice with phosphate-buffered saline (PBS). Next, the cells were gently resuspended in Annexin V binding buffer, incubated with Annexin V-FITC/PI in the dark for 15 min and analyzed using flow cytometry.

### Caspase activity assay

The SKOV-3 cells (1×10^6^) were incubated without or with shikonin (16 μM). The cells were harvested at 0, 12, 24, 48 and 72 h, washed with PBS and pelleted. The supernatant was aspirated, cell lysis buffer was added at 0.5 ml/1×10^6^ cells and then the cells in the lysis buffer were incubated on ice for 10 min. Reaction buffer containing 5 μl dithiothreitol, 5 μl DEVD-AFC amino acid substrate and 380 μl H_2_O was added to each aliquot of cell lysate and the mixtures were incubated at 37°C for 1 h. The fluorescence emitted by the cleaved substrates was determined using a spectrofluorometer at an absorbance of 400 nm for excitation and 505 nm for emission. One unit of enzyme activity corresponds to the activity required to cleave 1 mg of substrate in 1 min at 37°C.

### Migration assay

The SKOV-3 cells were plated onto the upper membrane of a Transwell unit (8-μm pore size; Merck Millipore, Darmstadt, Germany) at a density of 4×10^5^ cells/well. The cells were exposed to various concentrations of shikonin (0, 4, 8 and 16 μmol/l) and incubated for 24 h. Any non-migrated cells on the upper membrane were removed using a cotton swab, while the migrated cells (located on the lower surface of the Transwell filters) were fixed for 5 min in methanol, stained with 0.1% crystal violet, eluted with 33% ethylic acid and measured at an absorbance of 480 nm to obtain the optical density values.

### Western blot analysis

The SKOV-3 cells were harvested and lysed for 1 h on ice in lysis buffer [50 mM Tris-Cl (pH 7.4), 1% NP-40, 0.25% sodium deoxycholate, 150 mM NaCl, 1 mM EDTA, 1 mM PMSF, 1 mM Na_3_VO_4_, 1 mM NaF, mammalian protease inhibitor cocktail and phosphatase inhibitor cocktail; Roche Diagnostics, Indianapolis, IN, USA]. The caspase protein concentrations were determined by performing a bicinchoninic acid assay. The lysate was centrifuged at 16,000 × g and 4°C for 10 min, and then equal quantities of total protein were mixed with loading buffer, boiled for 5 min and subjected to electrophoresis on a 10% SDS-polyacrylamide gel. Subsequently, the proteins were electrotransferred onto polyvinylidene difluoride membranes. Subsequent to blocking with Tris-buffered saline (TBS) containing 5% skimmed milk at room temperature, the membranes were incubated for 2 h at room temperature with polyclonal rabbit anti-human caspase-3,-8 and -9 (1:1,000; Cell Signaling Technology, In., Danvers, MA, USA) primary antibodies in TBS. Following three 10-min washes in TBS, the membranes were incubated with a diluted horseradish peroxidase-labeled secondary antibody for 1 h and after an additional three washes, the protein expression levels were detected using an enhanced chemiluminescence kit, according to the manufacturer’s instructions (Bio-Rad Laboratories, Hercules, CA, USA).

### Statistical analysis

Data are presented as the mean ± standard deviation of the results from three independent experiments. Differences were assessed by the two-tailed Student’s t-test. Statistical analyses were performed using SPSS version 16.0 software (SPSS, Inc., Chicago, IL, USA). All graphs were obtained using Microsoft Office Excel 2010 software (Microsoft Research, Redmond, WA, USA). P<0.05 was considered to indicate a statistically significant difference.

## Results

### Inhibition of cell growth by shikonin

**Figure 1:**
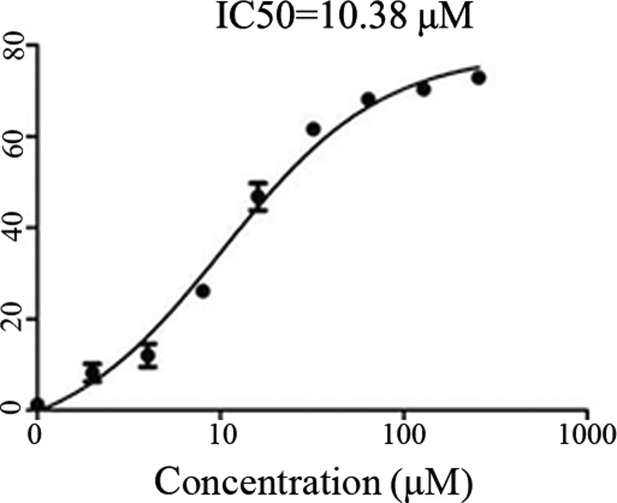
Inhibitory effect of shikonin on the growth of SKOV-3 cells. IC_50_, half maximal inhibitory concentration.

To determine the inhibitory effects of shikonin on the growth of cultured SKOV-3 cells and to ascertain the viability of SKOV-3 cells in the presence of shikonin, a CCK-8 assay was performed. Treating the SKOV-3 cells with increasing concentrations of shikonin identified 10.38 μmol as the half maximal inhibitory concentration of shikonin (Fig. 1).

### Shikonin induces the apoptosis of SKOV-3 cells

**Figure 2:**
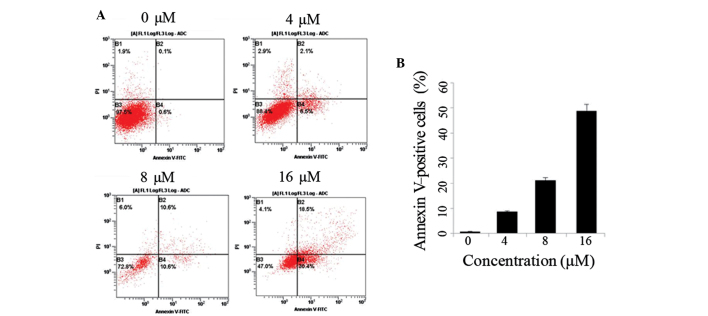
Shikonin induces cell death in SKOV-3 cells. (A) SKOV-3 cells were treated with dimethyl sulfoxide (DMSO; control) or 0, 4, 8 or 16 μM shikonin for 48 h, followed by use of an Annexin V-FITC binding assay. Cell death was measured by Annexin-V and PI staining, and flow cytometry; data are presented as cytograms. Viable cells were negative for Annexin-V and PI staining (lower left quadrant), early-stage apoptotic cells were positive for annexin-V staining, but negative for PI staining (lower right quadrant), and late-stage apoptotic cells were positive for Annexin-V and PI staining (upper right quadrant). (B) The percentage of Annexin V-stained cells following treatment with with DMSO (control) or 0, 4, 8 or 16 μM shikonin. Data are presented as the mean ± standard deviation (n=3). ^*^P<0.05 vs. control group. FITC, fluorescein isothiocyanate; PI, propodium iodide.

The SKOV-3 cells were treated with various concentrations of shikonin for 48 h (Fig. 2A) and the percentage of apoptotic cells present following treatment is indicated in Fig. 2B. Increasing concentrations of shikonin were associated with increased levels of SKOV-3 cell apoptosis.

### Effect of shikonin on caspase activation

**Figure 3:**
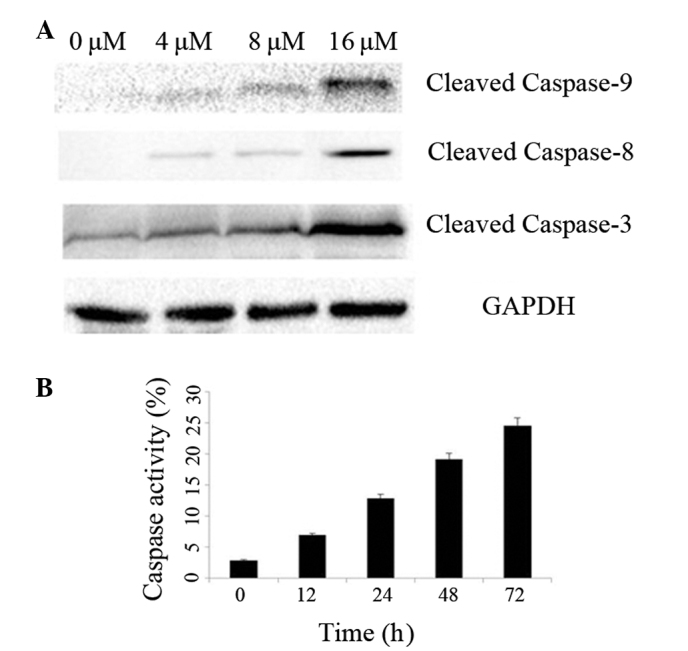
Shikonin activates caspases-3, -8 and -9 in SKOV-3 cells. (A) Western blot analysis demonstrating the effect of treatment with various concentrations of shikonin (0, 4, 8 and 16 μM) for 48 h on the expression of cleaved caspase-3, -8, and 9 (n=3). GAPDH was used as the internal control. (B) The activity of caspase-3 protease during shikonin-induced apoptosis (0, 12, 24, 48 and 72 h) following treatment with 16 μM shikonin. Data are presented as the mean ± standard deviation of three independent experiments.

The changes in caspase-3, -8 and -9 protein expression levels in response to shikonin administration were determined by performing western blot analysis (Fig. 3A) and it was found that the levels of cleaved caspase-3, -8 and -9 were increased following treatment with shikonin for 48 h. The activity of caspase-3 protease increased during shikonin-induced apoptosis (Fig. 3B)

### Effect of shikonin on the motility and invasion of SKOV-3 cells

**Figure 4:**
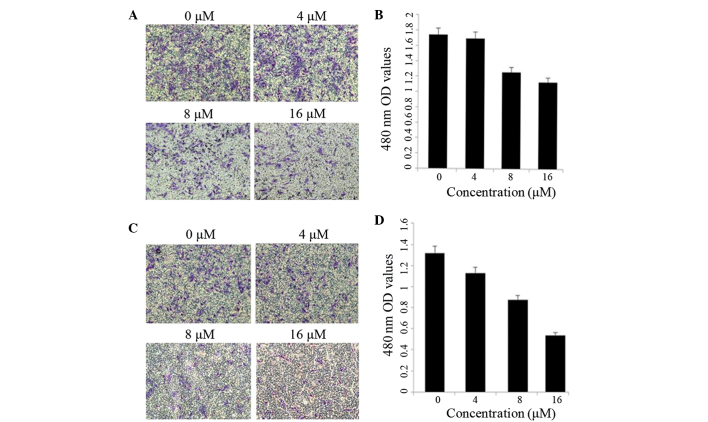
Shikonin dosage effects on SKOV-3 cell migration and invasion. (A) SKOV-3 cells were treated with dimethyl sulfoxide (DMSO; control) or 0, 4, 8 or 16 μM shikonin for 12 h, followed by a Transwell migration assay. (B) OD values of 33% ethylic acid eluent indicating the quantity of migrated cells. (C) SKOV-3 cells were treated with DMSO (control) or 0, 4, 8 or 16 μM shikonin for 24 h, followed by use of a Transwell invasion assay. (D) OD values of 33% ethylic acid eluent indicating the quantity of invasive cells. OD, optical density.

Treatment of the SKOV-3 cells with increasing concentrations of shikonin for 12 h resulted in a dose-dependent decrease in cell migration (Fig. 4A and B). Furthermore, the SKOV-3 cells that underwent longer treatment periods with shikonin (24 h) also exerted a dose-dependent inhibitory effect on cell invasion (Fig. 4C and D).

### Effect of shikonin on the activity and protein expression levels of Src and FAK in SKOV-3 cells

**Figure 5:**
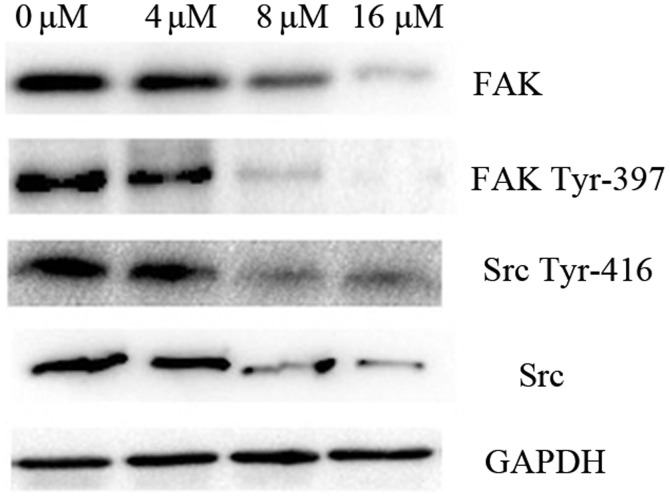
Effect of Shikonin on the expression of FAK, Tyr-397 phospho-FAK, Src and Tyr-416 phospho-Src protein in SKOV-3 cells. Western blot analysis demonstrated inhibition of FAK and Src protein expression levels following treatment with dimethyl sulfoxide (control) or 0, 4, 8 or 16 μM shikonin. FAK, focal adhesion kinase; Src, proto-oncogene tyrosine protein kinase Src.

The activity of Src and FAK in the SKOV-3 cells was measured by performing western blot analyses of Tyr-397-phosphoryated FAK and Tyr-416-phosphoryated Src. The activity of Src and FAK decreased in response to treatment with shikonin in a dose-dependent manner. Additionally, the expression of Src and FAK was decreased in the SKOV-3 cells in response to treatment with shikonin (Fig. 5). These results indicate that shikonin may be crucial in the downregulation of FAK expression.

## Discussion

Shikonin, a novel compound isolated from the Chinese herbal therapeutic agent Zicao, has been demonstrated to exhibit anticancer activity (16,17). Previous studies have indicated that shikonin inhibits tumor formation, carcinogenesis and metastasis, predominantly by inhibiting the proliferation and induction of apoptosis in tumor cell lines (18,19). Various molecular targets have been associated with shikonin-induced apoptotic cell death (20,21), however, the mechanism by which shikonin exhibits its anticancer activity is poorly understood.

In the present study, a potential therapeutic pathway was identified by which shikonin appears to target ovarian cancer cells by inhibiting growth and inducing apoptosis. Shikonin has previously been demonstrated to exhibit an inhibitory effect on human colorectal carcinoma COLO 205 cells via the induction of apoptotic cell death, accompanied by the upregulation of p27 and p53, and the downregulation of B-cell lymphoma (Bcl)-2 and Bcl-extra large (22). Furthermore, shikonin induces HeLa cell death via caspase-3 activation (23). Similarly, the present study detected caspase activation in shikonin-induced ovarian cell apoptosis. Furthermore, the extrinsic death receptor pathway and the intrinsic mitochondrial pathway appears to be involved in shikonin-induced apoptosis, indicating that the two pathways may interact with and amplify each other in the process of activating effector caspases, such as caspase-3 (23).

Additionally, the present study identified that shikonin decreases ovarian cancer cell migration and invasion; this was demonstrated by the treatment of SKOV-3 cells with various concentrations of shikonin, causing a reduction in the motility of the ovarian cancer cells. A shikonin derivative, β-hydroxyisovalerylshikonin, inhibits the PTK activities of epidermal growth factor and viral-Src receptors (24). Src and Src-family PTKs are regulatory proteins that play key roles in cell differentiation, motility, proliferation and survival (25). Src protein inhibition occurs via targeting of its phosphorylation sites; the Src phosphorylation sites initially described include the autophosphorylated activation site at Tyr 416. In agreement with the aforementioned previous studies, the present study demonstrated that shikonin inhibits Src activity in SKOV-3 cells.

FAK, a non-receptor PTK, is a key component of focal adhesion sites, particularly in the promotion, spread, migration and transmission of anchorage-dependent anti-apoptotic signals (26). FAK is activated following the engagement of adjacent integrin molecules or the stimulation of transmembrane receptors (27,28); and this activation occurs via tyrosine autophosphorylation, as well as via phosphorylation by other PTKs, including Src family kinases (29,30) and the insulin receptor (31). Tyr-397 autophosphorylation is an important step for promoting the biological function of FAK, and Tyr-576 and -577 phosphorylation by Src increases FAK activity (32). Previously, increased protein expression levels of FAK have been identified in various types of ovarian cancer, rendering FAK a potentially valuable target for therapeutic intervention (15). Furthermore, increased FAK expression and activity have previously been correlated with malignant or metastatic disease and poor patient prognosis (33). FAK is regulated by growth factor receptor stimulation and acts as a signaling intermediate that is recruited to focal adhesions immediately following integrin activation, therefore, the present study determined FAK activity in response to the presence of various concentrations of shikonin by analyzing the level of FAK autophosphorylation. In the SKOV-3 cells, shikonin decreased FAK activity in a dose-dependent manner. The present study additionally reported that shikonin appears to inhibit the metastasis of ovarian cancer cells; it is proposed that shikonin may mediate this metastatic effect by decreasing the activity and expression of FAK.

In conclusion, shikonin was shown to induce apoptosis and decrease cellular migration in ovarian cancer cells. In addition, shikonin decreased FAK activity in a dose-dependent manner. Therefore, shikonin requires consideration as a candidate agent for the prevention and treatment of human ovarian cancer.
